# Systematic Identification of MACC1-Driven Metabolic Networks in Colorectal Cancer

**DOI:** 10.3390/cancers13050978

**Published:** 2021-02-26

**Authors:** Jan Lisec, Dennis Kobelt, Wolfgang Walther, Margarita Mokrizkij, Carsten Grötzinger, Carsten Jaeger, Katharina Baum, Mareike Simon, Jana Wolf, Nicola Beindorff, Winfried Brenner, Ulrike Stein

**Affiliations:** 1 Medical Department of Hematology, Oncology, and Tumor Immunology, and Molekulares Krebsforschungszentrum (MKFZ), Chariteé–Universitaätsmedizin Berlin, 13353 Berlin, Germany; jan.lisec@bam.de (J.L.); carsten.jaeger@charite.de (C.J.); 2 German Cancer Consortium, Deutsches Krebsforschungzentrum (DKFZ), 69120 Heidelberg, Germany; Dennis.Kobelt@mdc-berlin.de (D.K.); wowalt@mdc-berlin.de (W.W.); margarita.mokrizkij@mdc-berlin.de (M.M.); Carsten.Groetzinger@charite.de (C.G.); Winfried.Brenner@charite.de (W.B.); 3 Department 1.7 Analytical Chemistry, Federal Institute for Materials Research and Testing (BAM), 12489 Berlin, Germany; 4 Experimental and Clinical Research Center, Charité–Universitätsmedizin Berlin, and Max-Delbrück-Center for Molecular Medicine in the Helmholtz Association, Translational Oncology of Solid Tumors, 13125 Berlin, Germany; 5 Department of Hepatology and Gastroenterology, Charité–Universitätsmedizin Berlin, 13353 Berlin, Germany; 6 Hasso Plattner Institute, Digital Engineering Faculty, University of Potsdam, 14482 Potsdam, Germany; Katharina.Baum@hpi.de; 7 Max Delbrück Center for Molecular Medicine in the Helmholtz Association, Mathematical Modeling of Cellular Processes, 13125 Berlin, Germany; Mareike.Simon@mdc-berlin.de (M.S.); jana.wolf@mdc-berlin.de (J.W.); 8 Berlin Experimental Radionuclide Imaging Center (BERIC), Charité–Universitätsmedizin Berlin, 13353 Berlin, Germany; Nicola.beindorff@charite.de; 9 Department of Nuclear Medicine, Charité–Universitätsmedizin Berlin, 13353 Berlin, Germany

**Keywords:** MACC1, cancer metabolism, metabolic profiling, metabolic networks, colorectal cancer

## Abstract

**Simple Summary:**

We aimed at the systematic identification of MACC1-driven metabolic networks in colorectal cancer. By this systematic analysis, our studies revealed new insights into MACC1-caused metabolomics phenotypes: (i) MACC1 fosters metastasis by rewiring glucose and glutamine metabolism, (ii) MACC1 increases glucose use by enhanced surface GLUT1; (iii) MACC1 increases glutamine and pyruvate use by enhanced uptake, and (iv) MACC1 reduces glutamine flux but has minor effects on pyruvate flux. Therefore, MACC1 is an important regulator of cancer metabolism.

**Abstract:**

MACC1 is a prognostic and predictive metastasis biomarker for more than 20 solid cancer entities. However, its role in cancer metabolism is not sufficiently explored. Here, we report on how MACC1 impacts the use of glucose, glutamine, lactate, pyruvate and fatty acids and show the comprehensive analysis of MACC1-driven metabolic networks. We analyzed concentration-dependent changes in nutrient use, nutrient depletion, metabolic tracing employing ^13^C-labeled substrates, and in vivo studies. We found that MACC1 permits numerous effects on cancer metabolism. Most of those effects increased nutrient uptake. Furthermore, MACC1 alters metabolic pathways by affecting metabolite production or turnover from metabolic substrates. MACC1 supports use of glucose, glutamine and pyruvate via their increased depletion or altered distribution within metabolic pathways. In summary, we demonstrate that MACC1 is an important regulator of metabolism in cancer cells.

## 1. Introduction

Redirection of energy metabolism is an emerging hallmark in cancer [1,2,3,4], often induced by oncogenes that are involved in programming the core hallmarks of cancer [5,6]. Exploiting oncogene-dependent metabolic alterations may open new options in personalized treatment.

Various nutrients, such as glucose, glutamine, lactate, pyruvate and fatty acids contribute to the survival of cancer cells and the dependence of cancer cells on certain nutrients is well known [7,8,9,10,11]. The main nutrient is glucose supporting not only cell proliferation, growth and survival, but also enhances invasion and metastatic potential [12,13]. Thus, the metabolic switch from respiration to the rather inefficient glycolysis to generate ATP, known as Warburg effect, is facilitated. Since glutamine use of cancer cells is known for several tumor entities, its targeting is effective in pancreatic, breast and colon cancers [14,15,16]. Lactate is known as key substrate used by glucose-deprived cancer cells to meet their energy demands [17,18]. Pyruvate correlates with increased cell proliferation and tumor aggressiveness, e.g., in breast cancer [19], and is associated with resistance to metabolic drugs [2]. Fatty acid use by glucose-deprived cancer cells promotes growth of ovarian cancer cells in vitro and in vivo [20].

The gene Metastasis-associated in colon cancer 1 (MACC1) was newly discovered in our group and is a key regulator of metastasis as well as a prognostic and predictive marker in many tumor entities [21,22,23,24,25,26,27,28]. However, its role in cancer metabolism is underinvestigated. MACC1 increased the Warburg effect via elevated expression of key glycolytic enzymes [29,30] in gastric and hepatocellular cancers, which is linked to trastuzumab resistance due to increased PI3K/AKT signaling [31]. In hepatocellular carcinoma [32] and in gastric cancer [33] MACC1 expression positively correlates with expression of 6-phosphofructo-2-kinase/fructose-2,6-bisphosphatase (PFKFB2) and of fatty acid synthase (FASN). Furthermore, MACC1 regulates transporters of the plasma membrane such as Na(+)/H(+) exchanger-1 (NHE1) [34] and MCT1 [35]. However, the role of MACC1 in the metabolism of colorectal cancer (CRC) is unknown.

Here, we carried out a thorough analysis of MACC1-dependent metabolic pathways by investigating the impact of MACC1 on the cellular metabolism of nutrients such as glucose, glutamine, lactate, pyruvate and fatty acids. We analyzed environment-dependent changes of nutrient use, metabolic tracing by ^13^C-labeled substrates and performed in vivo studies to better reveal the role of MACC1 in rewiring and in adaptation of CRC metabolism under stressed conditions.

## 2. Materials and Methods

### 2.1. Cell Culture and Derivative Cell Lines

All cell lines were obtained from ATCC (Manassas, VA, USA) and grown at 37 °C, 5% CO_2_. SW620, HCT116, HT29, HCT15 cells were grown in DMEM without glucose, glutamine, and phenol red (Gibco, Waltham, MA, USA, A14430) supplemented with 2 mM or 10 mM glucose (Sigma, St. Louis, MO, USA), 2 mM glutamine (Gibco) and 10% FBS (Bio&Sell, Feucht, Germany). SW480 cells were grown in RPMI-1640 (Gibco) supplemented with 10% FBS (Bio&Sell). Cells were kept sub-confluent and were passaged twice per week.

SW620 and HCT116 cells with stable MACC1 knockdown by stable shRNA expression and SW480 cells stably overexpressing MACC1 were generated as described previously [21]. SW620 cells with MACC1 knockdown and co-expression of luciferase reporter were generated as described previously [36].

### 2.2. Promoter Activity

For analysis of promoter activity 7 × 10^4^ HCT116 cells/well were seeded on a 24-well plate with complete DMEM without glucose, glutamine, phenol red (Gibco), supplemented with 10 mM glucose (Sigma), 2 mM glutamine (Gibco), 10% FBS (Bio&Sell). After 12 h medium was changed to DMEM supplemented with 0 mM, 2 mM or 10 mM glucose (Sigma), respectively, and 10% FBS (Bio&Sell). Twelve hours later HCT116 cells were transfected as described previously [37]. Forty-eight hours after transfection cells were analyzed by using the dual luciferase reporter assay (Promega, Fitchburg, WI, USA) and values of firefly luciferase were normalized to Renilla luciferase values. 

### 2.3. Quantitative qPCR

RNA was isolated using an RNA extraction kit (Roboklon, Berlin, Germany) and 1 µg of total RNA was used for cDNA synthesis. All primers were pre-designed KiCqStart^®^ SYBR^®^ Green Primers (Sigma), except for MACC1 and human microsatellite DNA (both BioTeZ, Berlin, Germany). The primers for MACC1 were as described in (Stein et al., 2009 [21]). The primers for human microsatellite DNA: fw 5′-GGGATAATTTCAGCTGACTAAACAG-3′; rv 5′-AAACGTCCACTTGCAGATTCTAG-3′. The primers for 28S rRNA: fw 5′-GTTCACCCACTAATAGGGAACGTGA-3′; rv 5′-GGATTCTGACTTAGAGGCGTTCAGT-3′.

### 2.4. Western Blotting

Western blotting was performed as described previously (Stein et al., 2009 [21]) using the following antibodies: MACC1 (HPA020103, Sigma), GLUT1 (ab115730, Abcam, Cambridge, UK), β-actin (A19778, Sigma).

### 2.5. Cell Viability

For cell viability 0.3 × 10^6^ SW620 and HCT116 cells/well were seeded on a 6-well plate with DMEM without glucose, glutamine, phenol red (Gibco), supplemented with 10 mM glucose (Sigma), 2 mM glutamine (Gibco) and 10% FBS (Bio&Sell). SW480 cells (0.1 × 10^6^ cells/well, since SW480 are larger compared to SW620 we seeded less cells from SW480 to cover a comparable plating area compared to the smaller SW620) were seeded on a 6-well plate with RPMI-1640 (Gibco) supplemented with 10% FBS (Bio&Sell). Twelve hours after cell seeding the medium was changed on the corresponding medium. Cells were treated with 10 µM WZB117 (Millipore, Dundee, UK), 0.25 mM 6-Diazo-5-oxo-L-norleucin (DON, Sigma), 10 µM cPEPCK inhibitor (Axon Medchem, Groningen, Netherlands) and 50 nM rotenone (Sigma). WZB117 and cPEPCK inhibitors were dissolved in DMSO. DON and rotenone were dissolved in complete DMEM. Forty-eight hours after treatment cells were trypsinized and counted using Neubauer hemocytometer (Celeromics, Paterna, Spain).

### 2.6. Conjugation of Palmitate to Bovine Serum Albumine (BSA)

For conjugation of palmitate (Sigma) with BSA (Sigma), 538.4 mg ultra-low fatty acid BSA was diluted in 12 mL autoclaved, 37 °C warm 150 mM NaCl, followed by incubation at 37 °C till BSA is dissolved. Then, 9.18 mg palmitate was diluted in 6.6 mL of warm 150 mM NaCl and then heated at 70 °C with stirring till the solution was clear. Thereafter, 8.25 mL warm BSA solution was transferred to a new tube and palmitate solution was added slowly to BSA solution. The conjugate of palmitate to BSA was stirred for 1 h at 37 °C. Then, 1.65 mL 150 mM NaCl solution was added to the conjugate and pH was adjusted to 7.4 with 1 M NaOH. The conjugate solution was aliquoted and stored at −20 °C. BSA vehicle was prepared by mixing 3 mL BSA solution with 3 mL 150 mM NaCl. BSA vehicle was aliquoted and stored at −20 °C. The molar ratio of conjugated palmitate:BSA was as 6:1. Prior cell treatment with palmitate:BSA conjugate, both BSA and palmitate were thawed at 37 °C for 10 min.

### 2.7. Metabolic Tracing

For ^13^C_6_ glucose and ^13^C_5_ glutamine (10 mM glucose/2 mM glutamine) tracing experiments, 0.25 × 10^6^ SW620 shcntl and SW620 shMACC1 cells/well were seeded into 6-well plate with DMEM without glucose, glutamine, phenol red (Gibco), supplemented with 10 mM glucose (Sigma), 2 mM glutamine (Gibco) and 10% FBS (Bio&Sell). After 24 h medium was changed to DMEM without glucose, glutamine, phenol red (Gibco), supplemented with 10 mM ^13^C_6_ glucose (Sigma), 2 mM glutamine (Gibco) and 10% FBS (Bio&Sell) (for glucose) or to DMEM without glucose, glutamine, phenol red (Gibco), supplemented with 10 mM glucose (Sigma), 2 mM ^13^C_5_ glutamine (Gibco) and 10% FBS (Bio&Sell) (for glutamine). Cells were incubated with labeled substrate for 24 h. Then, medium and cell fractions were taken, separately. Cells were washed with phosphate buffered saline (PBS). Then, 150 µL of −20 °C cold methanol (Sigma) was added and incubated for 5 min at room temperature. Then, 150 µL of sterile, distilled water (Gibco) was added, cells were scraped and transferred to a 2 mL tube, and 1.3 mL chloroform (Sigma) was added. The lysate was vortexed and placed on ice for 30 min. Then, 300 µL of sterile distilled water (Gibco) was added and the mixture was centrifuged at 3000× *g*, 4 °C for 20 min. The aqueous phase was transferred into a new tube and stored at −80 °C till analysis.

For ^13^C_3_ pyruvate tracing experiments, 0.25 × 10^6^ SW620 shcntl and SW620 shMACC1 cells/well were seeded into 6-well plate with DMEM without glucose, glutamine, phenol red (Gibco), supplemented with 10 mM glucose (Sigma), 2 mM glutamine (Gibco) and 10% FBS (Bio&Sell) and allowed to attach for 12 h. Then, medium was changed to DMEM without glucose, glutamine, phenol red (Gibco), supplemented with 2 mM glutamine (Gibco), 10 mM pyruvate (Gibco) and 10% FBS (Bio&Sell). Cells were cultivated for 24 h, medium was changed to DMEM without glucose, glutamine, phenol red (Gibco), supplemented with 2 mM glutamine (Sigma), 10 mM ^13^C_3_ pyruvate (Sigma) and 10% FBS (Bio&Sell). Cells were incubated in the presence of ^13^C_3_ pyruvate (Sigma) for 24 h and metabolite extraction was performed as described above.

### 2.8. GC-MS

Samples were dried in a SpeedVac® vacuum concentrator (VWR) and derivatized with 15 µL methoxylamine HCl (MeOX)-pyridine (Sigma) and 60 µL N-trimethylsilyl-N-methyl trifluoroacetamide (MSTFA, Sigma). Fatty acid methyl esters (FAMEs, Sigma) were spiked as retention index markers in total volume of 2 µL to the derivatization mixture. Samples were processed automatically in a pipetting robot and 1 µL of a sample with a split ratio of 1:100 was run at the GC-APCI-MS (Agilent 7890 GC coupled to Bruker impact II MS). The analysis of the peaks was performed using several publicly available R packages (HiResTEC, MetabolomicsBasics, CorrectOverloadedPeaks and InterpretMSSpectrum; [38,39,40,41]. An in-house library was used to confirm each metabolite identity. The ion intensity of all isotopologues of the molecular ion peak which contain carbon of biological origin were extracted. These mass isotopologue distributions (MIDs) were corrected for natural abundant ^13^C as well as overlaying for MIDs due to proton loss using in-house R-scripts.

Each peak for metabolites in an in-house library was checked manually. In case of a significant retention time (RT) shift, the RT was adjusted manually, or in case of a co-elution a different fragment was selected.

### 2.9. Flow Cytometry

SW480 and SW620 cells were collected, fixed with 4% paraformaldehyde and stained with anti-GLUT1 mouse antibody (R&D Systems, Minneapolis, MN, USA). For the negative control, cells were stained with an appropriate isotype control. Flow cytometry was performed at BD LSRFortessa™ cell analyzer (BD Biosciences, Franklin Lakes, NJ, USA). For signal acquisition 10,000 cells were used and data was presented as net mean fluorescence intensity (net MFI), depicted as number of Fluorescein isothiocyanate (FITC)-positive cells multiplied by FITC geometric mean. The data was analyzed with FlowJo^®^ software (FLOWJO, LLC, Ashland, OR, USA).

### 2.10. Oxygen Consumption Rate (OCR)and Extracellular Acidification Rate (ECAR)

The Seahorse XFp Cell Energy Phenotype Test kit (Agilent, Santa Clara, CA, USA) and the Seahorse XFp Cell Mito Stress Test Kit (Agilent) were used to assess glycolytic and mitochondrial functionality. On the day prior to the assay, 8 × 10^4^ SW620 cells/well and 3 × 10^4^ SW480 cells/well were seeded in DMEM without glucose, glutamine, phenol red (Gibco), supplemented with 10 mM glucose (Sigma), 2 mM glutamine (Gibco) and 10% FBS (Bio&Sell); or with RPMI (Gibco) supplemented with 10% FBS (Bio&Sell), respectively. For metabolic measurements, the Seahorse XFe96 Analyzer (Agilent) was used and all data were analyzed by Seahorse Wave software (Agilent). All measurements were normalized by protein content.

### 2.11. ATP Measurements

Mitochondrial ATP measurements were extrapolated from oxygen consumption rate values upon oligomycin and carbonyl cyanide-4 (trifluoromethoxy) phenylhydrazone (FCCP) treatments. Total ATP measurement was performed by CellTiter-Glo^®^ Luminescent cell viability assay (Promega) and luminescence was normalized by blank medium containing wells without cells.

### 2.12. Glucose, Glutamine, Pyruvate and Lactate Assays

Glucose concentration in cell growth medium was determined by GC-MS. Glutamine concentration in cell growth medium was analyzed by EnzyChrom^TM^ glutamine assay kit (EnzyChrom, San Jose, CA, USA). EnzyChrom™ pyruvate assay kit (EnzyChrom) was used for determination of pyruvate concentration in cell growth medium and EnzyChrom^TM^ lactate assay kit (EnzyChrom) was employed for analysis of lactate concentration in cell growth medium. For all these assays blank values were subtracted from values for standards and samples. Metabolite concentrations were determined from the slope of the respective standard curves.

### 2.13. Animal Experiments

The mouse experiments were performed in accordance with guidelines of the United Kingdom Co-ordinated Committee on Cancer Research (UKCCCR) and approved by the institutional review board of the Charité–Universitätsmedizin Berlin, Germany, approval number AA3/03/45.

For intrasplenic tumor cell injection, 6–8-week-old SCID/beige female mice (Charles River, Wilmington, MA, USA) were used (epo GmbH, Berlin). Briefly, 3 × 10^6^ SW620/luc shcntl and SW620/luc shMACC1 cells were injected into the spleen of anesthetized mice (35 mg/kg Hypnomidate^®^; Jassen-Cilag, Neuss, Germany). The tumor and liver metastases were allowed to form for 28 days before imaging with positron emission computed tomography (PET)/MRI. Tumor growth and metastasis formation was monitored by bioluminescence imaging (BLI) using the NightOWL LB 981 imaging system (Berthold Technologies, Bad Wildbad, Germany). For BLI, anesthetized mice (isoflurane) were injected intraperitoneally with 150 mg/kg D-luciferin (Biosynth, Staad, Switzerland) and tumor growth and metastasis formation in livers was imaged and quantified by WinLight (Berthold Technologies) and ImageJ 1.48v.

### 2.14. PET/MRI

Five female SW620 shcntl and four female SW620 shMACC1 SCID/beige mice were used for tomographic imaging at the Berlin Experimental Radionuclide Imaging Center (BERIC), Charité–Universitätsmedizin Berlin, using the dedicated small animal 1 Tesla nanoScan PET/MRI (Mediso, Budapest, Hungary). Isoflurane anaesthetized mice were tail vein injected either with 80 µl contrast agent Primovist™ (1:5 diluted with 0.9% sodium chloride) for contrast-enhanced magnetic resonance imaging (MRI) or 200 µl of ^18^F-fluoro-deoxyglucose (^18^F-FDG, 6.5–15.6 MBq) for PET. In addition, two SW620 shcntrl and two SW620 shMACC1 mice were injected with 3.5–5.0 MBq ^18^F-fluoropropyl-glutamate (^18^F-glutamate) after 48 h for repeated PET scanning [42]. PET scans with ^18^F-FDG or with ^18^F-glutamate were performed for 20 min starting 1 h after tracer injection. The uptake of ^18^F-FDG or ^18^F-glutamate in the tumor tissue of the liver and spleen was determined by manual contouring of a volume of interest (VOI) of the PET image using PMOD 3.5 (PMOD Technologies Ltd., Zurich, Switzerland). Furthermore, uptake in the thigh muscle of the hind limb was taken as reference value to assess differences in tracer distribution volume between animals. The uptake value was computed from the 10 hottest voxels within the ^18^F-FDG- or ^18^F-glutamate positive lesions and calculated by normalizing the integrated activity of each VOI to the total injected activity/mL (%IA/mL). 

For cross-checking, the uptake of the tumor lesions was normalized to the uptake of the thigh muscle by the formula: tumor/muscle ratio = %IA/mL tumor/%IA/mL muscle. This was done to correct for potential differences in uptake caused by differences in the distribution volume, such as animal size and weight, or renal function in mice etc., which both influence tumor and muscle uptake.

Anatomic MRI scans were acquired using a T1-weighted 3D spoiled Gradient Echo sequence (T1 GRE 3D) at the following parameters: coronal as well as transverse sequentially, matrix 256 × 256 × 42 with dimensions 0.23 × 0.23 × 0.5 mm^3^, TR: 15 ms, TE: 2.9 ms, and a flip angle of 25°.

### 2.15. Immunohistochemistry

Cryosections (thickness of 10 µm, for DNA and RNA extraction; or 5 µm, for immunohistochemistry) of spleens (the tumor implantation site) and livers (the metastasis target organ) were prepared. Genomic DNA and total RNA was extracted by the DNA/RNA/Protein extraction kit (Roboklon). Immunohistochemical staining was performed using the anti-CK19 antibody (TA336845, OriGene, Rockville, MD, USA) at a dilution 1:200. CK19 protein was visualized using Dako DAB liquid (Agilent). Negative control experiments were performed by staining without primary antibody. Tissue sections were imaged with Zeiss Axioplan2 microscope and analyzed with AxioVision^®^ software (Oberkochen, Germany). 

### 2.16. Statistical Analysis

All statistical analyses were performed with GraphPad Prism version 6.01. For all statistical analyses two-sided, two-tailed Student’s *t*-test was used. The *p*-values of less than 0.05 were defined as statistically significant.

## 3. Results

### 3.1. MACC1 Enhances ^18^F-FDG and ^18^F-Glutamate Uptake In Vivo

**Figure 1:**
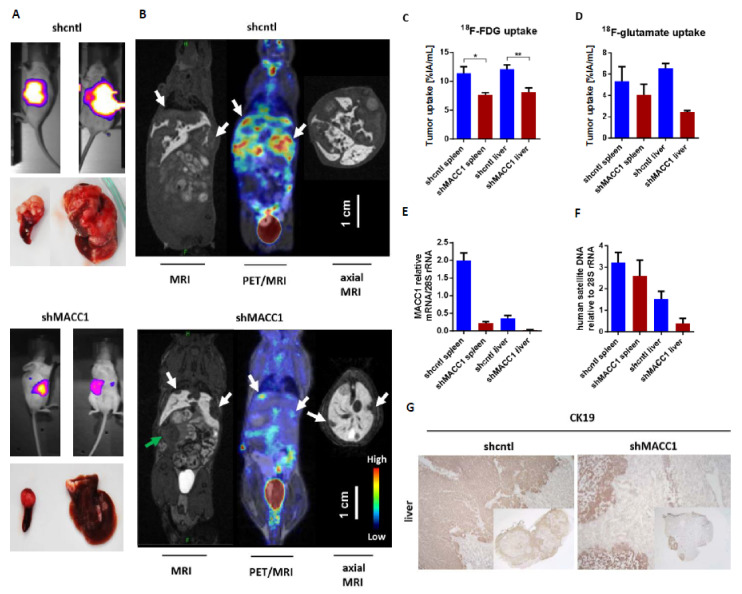
MACC1 enhances ^18^F-FDG and ^18^F-glutamate uptake in vivo. (**A**) Lateral and ventral bioluminescence imaging of SCID/beige mice 21 days post intrasplenic injection of SW620 shcntl or SW620 shMACC1 cells; transplanted tumor-bearing spleens and liver metastases of these animals are shown. (**B**) PET/MR images of liver metastases (white arrows) 60 min after intravenous injection of 9 MBq or 14 MBq ^18^F-FDG in SW620 shcntl or SW620 shMACC1 tumor-bearing SCID/beige mice. T1 GRE EXT 3D coronal MRI shows the metastases with hypointensity, clearly surrounded by normal, vital mouse liver tissue, represented by the hyperintensity of contrast agent Gadolinium™; fused MR and PET image; T1 GRE EXT 3D axial MRI of the liver. The primary tumor in the spleen is indicated by green arrow. In all 3 images scale bar is valid for MRI, PET/MRI and axial MRI. Four mice were imaged in the shcntl group, whereas five mice were imaged in shMACC1 group. (**C**) Quantification of ^18^F-FDG and (**D**) of ^18^F-glutamate uptake in the primary tumor in the spleen and in liver metastases. Four SW620 shcntl and five SW620 shMACC1 mice were analyzed for ^18^F-FDG measurements. Two mice per group were analyzed for ^18^F-glutamate measurements. Data represent mean values ± SEM, * *p* ≤ 0.05, ** *p* ≤ 0.01. (**E**) MACC1 mRNA expression and (**F**) quantification of human microsatellite DNA in primary tumor (spleen) and liver metastases. Data represent mean values ± SEM. (**G**) CK19-specific immunohistochemistry in cryosections from mouse liver with metastases of SW620 shcntl or SW620 shMACC1 cells. Brown staining indicates human tumor cells with human CK19 expression. Images: 10-fold magnification; inset depicting whole liver tissue: 0.8-fold magnification. CK19: cytokeratin 19. * *p* ≤ 0.05, ** *p* ≤ 0.01.

First, we tested, if MACC1 influences the uptake of the important nutrient glucose and glutamine in vivo. For performing metabolic substrate uptake studies, we intrasplenically injected ten SCID/beige mice with SW620 small hairpin (sh)cntl CRC cells which are endogenously high in MACC1, or with SW620 sh MACC1 cells as controls. The tumors were grown for 21 days. Tumor growth and metastasis formation in the liver were monitored by BLI. SW620 shcntl group mice had a higher tumor load and enhanced metastasis formation compared to the SW620 shMACC1 mice (Figure 1A).

To monitor uptake of glucose analog ^18^F-fluorodeoxyglucose (^18^F-FDG) as well as of glutamate analog ^18^F-fluoropropyl-glutamate (^18^F-glutamate) during in vivo metabolic studies, positron emission tomography (PET) was used. ^18^F-FDG was intravenously injected into SCID/beige mice bearing primary tumors and liver metastases as confirmed by MRI (Figure 1B). For the SW620 shcntl group a significantly higher ^18^F-FDG uptake of 48% in the primary tumor and 47% in liver metastases was measured, compared to the SW620 shMACC1 group (primary tumor: *p* = 0.025; liver metastases: *p* = 0.008; Figure 1B,C). The analysis of tumor/muscle ratio (% injected activity (IA)/mL tumor/%IA/mL muscle) revealed a higher ^18^F-FDG uptake of 35% for both primary tumor and liver metastases of the SW620 shcntl group compared to the SW620 shMACC1 group (primary tumor: *p* = 0.02; liver metastases: *p* = 0.022).

Two days after ^18^F-FDG imaging we injected^18^F-glutamate intravenously. We found increased ^18^F-glutamate uptake of 32% in primary tumor and 168% in liver metastases of the SW620 shcntl group compared to the SW620 shMACC1 group (Figure 1D). The tumor/muscle ratio of the SW620 shcntl group was elevated by 18% and 124% for primary tumor and liver metastases compared to the SW620 shMACC1 group. Uptake to the thigh muscle served as control, for which no significant differences in ^18^F-FDG and ^18^F-glutamate uptake between the SW620 shcntl and SW620 shMACC1 groups were observed (*p* = 0.526 and *p* = 0.377, respectively). This strengthens our hypothesis on MACC1-related differences in tumor uptake.

For molecular analysis primary tumors and liver metastases of the SW620 shcntl and SW620 shMACC1 groups were used. We detected high MACC1 expression in the SW620 shcntl group and knockdown of MACC1 expression in the SW620 shMACC1 group (Figure 1E). Furthermore, analysis of human satellite DNA revealed increased human cell load in mouse livers of the SW620 shcntl group compared to mouse livers of the SW620 shMACC1 group (Figure 1F). Immunohistochemical staining of mouse livers for human epithelial cell marker cytokeratin 19 (CK19), determined larger areas of CK19 positivity in the SW620 shcntl group compared to the SW620 shMACC1 group (Figure 1G). 

In conclusion, these data show that MACC1 increases formation of liver metastases, which is in line with earlier reports [21], and that this is accompanied by increased in vivo glucose and glutamate/glutamine uptake. This demonstrates an effect of MACC1 on cancer metabolism.

### 3.2. MACC1 Gene Expression Is Enhanced during High Glucose Abundance through Increased Uptake via GLUT1

**Figure 2:**
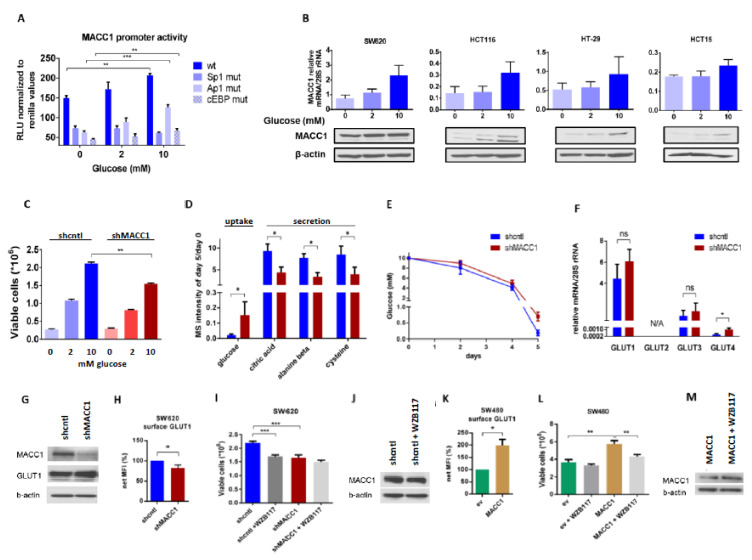
Surface GLUT1 is enhanced in MACC1 high cells. (**A**) MACC1 wt. promoter activity or activity with indicated mutated Sp1, Ap1, or cEBP binding sites was measured in HCT116 cells, treated with 0 mM, 2 mM and 10 mM glucose. (**B**) MACC1 mRNA and protein expression in SW620, HCT116, HT29 and HCT15 cells treated with 0 mM, 2 mM and 10 mM glucose. (**C**) Cell viability of SW620 shcntl and MACC1 shMACC1 (knockdown) cells treated with 0 mM, 2 mM and 10 mM of glucose. (**D**,**E**) GC-MS screening of growth medium metabolites after 5 days of culture of SW620 shcntl and SW620 shMACC1 cells grown in basal medium (supplemented with 10 mM glucose and 2 mM glutamine). (**D**) Levels of top differential metabolites, expressed relative to day 0 with fold-change > 2 and *p* < 0.05. (**E**) Gradual glucose depletion from (**D**) over period of 5 days. (**F**) GLUT1, GLUT2, GLUT3 and GLUT4 mRNA expression levels of SW620 shcntl and shMACC1 cells. (**G**) Total GLUT1 and, (**H**) GLUT1 surface protein expression in SW620 shcntl and shMACC1 cells. (**I**) Cell viability of WZB117 GLUT1 inhibitor treated SW620 shcntl and shMACC1 cells. (**J**) MACC1 expression of WZB117 treated SW620 shcntl cells. (**K**) GLUT1 surface expression in SW480/empty vector (ev) and SW480/MACC1 cells. (**L**) Cell viability of WZB117 treated SW480/ev and SW480/MACC1 cells. (**M**) MACC1 expression of WZB117 treated SW480/MACC1 cells. Data represent mean values ± SEM of at least three independent experiments; for GC-MS screening *n* = 3 technical replicates; * *p* ≤ 0.05, ** *p* ≤ 0.01, *** *p* ≤ 0.001.

To investigate the dependence of MACC1 expression on glucose availability, we measured the MACC1 promoter activity [37] in HCT116 cells supplied with increasing glucose amounts. Here, we observed a glucose-dependent increase in MACC1 promoter activity. Since the regulation of the MACC1 promoter by Sp1, Ap1 and cEBP was previously described, we analyzed the impact of these transcription factors on the glucose-dependent MACC1 regulation. The promoter activity was strongly reduced when one of these transcription factor binding sites was mutated. MACC1 promoter constructs with mutated Ap1 and cEBP binding sites were still responsive to increasing glucose concentrations. If the Sp1 binding site was mutated there was no increase of the promoter activity by glucose detectable anymore. Therefore, the glucose-mediated increase of MACC1 gene expression is at least partially mediated by the Sp1 binding site (Figure 2A). Moreover, higher glucose levels increased MACC1 mRNA and protein amount in SW620, HCT116, HT29 and HCT15 colorectal cancer cells (Figure 2B). Although the data did not reach statistical significance, the trend, especially at the protein level, is systematic and might indicate a biological relevance. As MACC1 protein expression was highest in SW620 among all cell lines tested, further experiments, unless indicated otherwise, were performed in SW620 cells.

Glucose is a well-known stimulus of expression of numerous genes. This often results in increased cell proliferation. Therefore, we were interested in whether MACC1, once being up-regulated by increased glucose availability, can further enhance glucose-dependent cell proliferation. To test this, we performed lentiviral shRNA-mediated knockdown of MACC1 in SW620 cells and treated these cells with increasing glucose concentrations.

As shown in Figure 2C, MACC1 enhanced cell proliferation in high and low glucose conditions, whereas in conditions without glucose MACC1 had no effect on cell proliferation. We hypothesized that the enhancement of cell proliferation by MACC1 may be reflected by altered consumption of nutrients from cell growth medium, mostly but not exclusively of glucose. Therefore, we monitored nutrients present in the growth medium of MACC1 control versus knockdown cells during a 5-day cultivation time using gas chromatography coupled mass spectrometry (GC-MS). We assumed that those nutrients, which enhance cell proliferation, should be consumed more rapidly. Only glucose was found to be differentially depleted, in part due to MACC1-mediated increased growth of shcntl cells (Figure 2D,E).

Next, we aimed to analyze the effect of MACC1 on expression or altered distribution of glucose transporters. At mRNA and protein levels (Figure 2F,G) there was only a significant difference in the lowly abundant GLUT4 glucose-specific transporter expression between MACC1 control and knockdown cells, but not in the most abundant GLUT1 transporter. However, staining of cells for surface GLUT1 revealed that in MACC1 knockdown cells surface GLUT1 was significantly reduced compared to control cells (Figure 2H). Treatment with GLUT1 inhibitor WZB117 significantly reduced proliferation of MACC1 control cells (Figure 2I). MACC1 down-regulation by shRNA had a similar effect (Figure 2I). In addition, treatment with WZB117 led to decreased MACC1 expression (Figure 2J). In SW480 cells, which have endogenously low MACC1 expression, overexpression of MACC1 led to increased surface GLUT1 and to enhanced cell proliferation (Figure 2K,L). Treatment of these cells with WZB117 markedly reduced cell proliferation, but did not reduce the CMV promoter driven MACC1 expression (Figure 2M).

In summary, our results indicate the presence of a positive feedback loop, where MACC1, once up-regulated by glucose, increases surface GLUT1, which in turn leads to increased glucose consumption and causes increased cell proliferation. Based on this, targeting of GLUT1 by inhibitors is more efficient in cells with high MACC1 expression.

### 3.3. MACC1 Reduces Mitochondrial Respiration and Spare Respiratory Capacity

**Figure 3:**
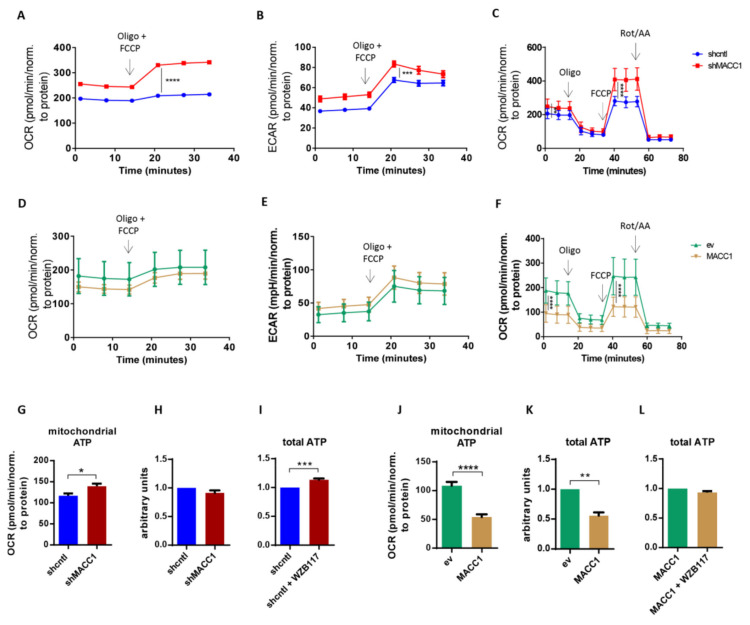
Decreases of mitochondrial respiration and spare respiratory capacity by MACC1. (**A**) Oxygen consumption rate (OCR) and (**B**) extracellular acidification rate (ECAR) of SW620 shcntl and SW620 shMACC1 cells treated with both oligomycin (Oligo) and FCCP. (**C**) OCR of cells as in (**A**,**B**), treated with Oligo/FCCP and rotenone/antimycin A (Rot/AA). (**D**) OCR and (**E**) ECAR of SW480 empty vector (ev) and MACC1 overexpressing (MACC1) cells treated with the mixture of Oligo/FCCP. (**F**) OCR of cells as in (**D**,**E**) treated with Oligo/FCCP and Rot/AA. (**G**) Mitochondrial and (**H**) total ATP in SW620 shcntl and SW620 shMACC1 cells without or, (**I**) with GLUT1 inhibitor WZB117. (**J**) Mitochondrial and (**K**) total ATP in SW480 ev and SW480 MACC1 cells without or, (**L**) with GLUT1 inhibitor WZB117. * *p* ≤ 0.05, ** *p* ≤ 0.01, *** *p* ≤ 0.001, **** *p* ≤ 0.0001. Data represent mean values ± SEM of at least three independent experiments.

Next, we investigated the impact of MACC1 on glycolysis and mitochondrial respiration as functional read-outs using a Seahorse analyzer. OCR and extracellular acidification rate (ECAR) demonstrated that SW620 shcntl cells were less energetic compared to SW620 shMACC1 cells (Figure 3A,B). Treatment with the ATP synthase inhibitor oligomycin and the mitochondrial uncoupler carbonyl cyanide-4-(trifluoromethoxy) phenylhydrazone (FCCP) resulted in an elevated increase in respiration in SW620 shMACC1 cells compared to SW620 shcntl cells (Figure 3A,C). Conversely, overexpressed MACC1 in SW480 cells tentatively lowered respiration upon oligomycin and FCCP treatment (Figure 3D,F). Knockdown of MACC1 resulted in increased ECAR in SW620 cells (Figure 3B), whereas MACC1 overexpression in SW480 cells was unable reverse this effect (Figure 3E).

Mitochondrial respiration is linked to mitochondrial ATP production. Mitochondria-derived ATP increased in SW620 shMACC1 cells compared to SW620 shcntl cells (Figure 3G). However, overexpression of MACC1 in SW480 cells, led to a reduction of mitochondria-derived ATP (Figure 3J). Mitochondria-derived ATP increased in SW620 shMACC1 cells, whereas total ATP remained unchanged, if SW620 shcntl and SW620 shMACC1 cells are compared (Figure 3H). Glucose was depleted faster from cell growth medium of SW620 shcntl cells compared to SW620 shMACC1 cells (Figure 2D,E). This clearly shows that reduced respiration and hence mitochondrial ATP production may serve as a compensation for increased glucose uptake. Indeed, treatment of SW620 shcntl cells with GLUT1 inhibitor WZB117 causes increased total ATP production (Figure 3I). In SW480 cells, MACC1 overexpression lowered total ATP (Figure 3K) and, WZB117 treatment of MACC1-overexpressing cells did not affect total ATP production (Figure 3L).

In summary, we show that MACC1 expression regulates mitochondrial respiration for ATP production, judged by respiratory capacity. 

### 3.4. MACC1 Enhances Glutamine Use in Glucose-Depleted Conditions

In this study, we investigated the effect of MACC1 on the use of alternative metabolic fuels, including glutamine, pyruvate, lactate and fatty acids, depending on the environmental conditions.

**Figure 4:**
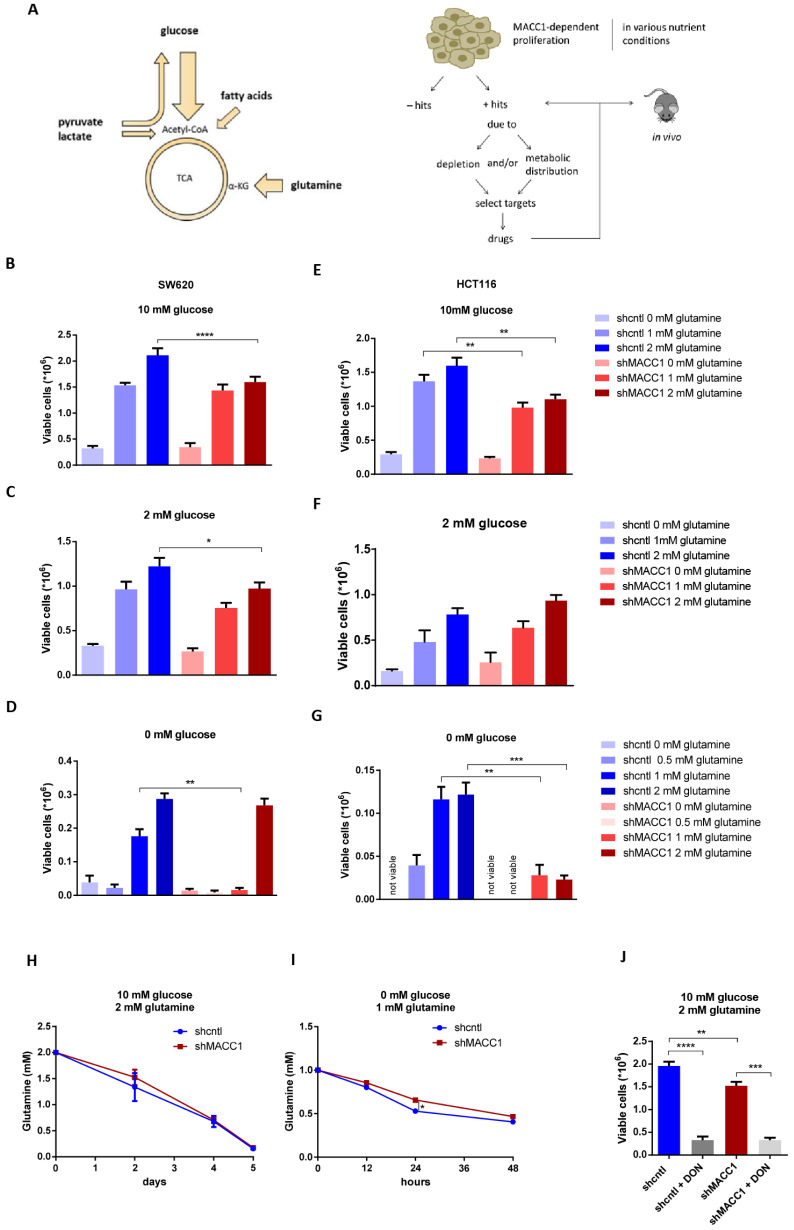
MACC1 impact on glutamine use. (**A**) Schematic representation of metabolic pathways fueled by indicated nutrients and workflow outline for identification of MACC1-dependent nutrient conditions influencing cell proliferation. MACC1-dependent cell proliferation is investigated under different nutrient conditions. Conditions by which MACC1 does not increase proliferation are considered to be negative hits. Conditions by which MACC1 supports cell proliferation are considered positive hits, for which then further uptake and ^13^C labeled substrate studies are performed. Depending on the results of uptake and ^13^C studies, targets and their drugs are selected and applied to positive hit conditions in vitro, followed by animal experiments. Cell viability of SW620 shcntl and SW620 shMACC1 cells treated with various glutamine concentrations (**B**) in high, (**C**) in low and (**D**) in no glucose conditions. (**E**–**G**) Cell viability of HCT116 shcntl and HCT116 shMACC1 cells treated with various glutamine concentrations (**E**) in high, (**F**) in low and (**G**) in no glucose conditions. Glutamine depletion from cell growth medium of SW620 shcntl and SW620 shMACC1 cells after (**H**) 5 days of culture in nutrient replete medium and (**I**) after 48 h of culture at indicated conditions. (**J**) Cell viability of SW620 shcntl and SW620 shMACC1 cells grown in basal medium and treated with DON, an inhibitor of glutamine-using enzymes. * *p* ≤ 0.05, ** *p* ≤ 0.01, *** *p* ≤ 0.001, **** *p* ≤ 0.0001. Data represent mean values ± SEM of at least three independent experiments.

We present the general outline of our studies in Figure 4A. First, we determined MACC1-dependent cell proliferation, assessed under various nutrient conditions. These conditions were considered to be positive hits. We investigated, how MACC1 increased the use of certain nutrients in positive hit conditions, by nutrient depletion studies and metabolite tracing using ^13^C-labeled metabolic substrates, by selection of targets, and by application of potential drugs in positive hit conditions (Figure 4A). Performing analyses according to this outline, the relation of MACC1 to glutamine use was assessed first.

Glutamine is the second most important nutrient after glucose used by cells to generate precursors necessary for cell growth and survival. Here we analyzed the relevance of glutamine for MACC1-dependent cell proliferation. A high glucose (10 mM), low glucose (2 mM) and no glucose (0 mM) environment combined with various glutamine concentrations was applied to SW620 shcntl, SW620 shMACC1 as well as to HCT116 shcntl and HCT116 shMACC1 cells. MACC1 had no effect on cell proliferation in these SW620 and HCT116 cells if glucose is abundant and glutamine is depleted (Figure 4B,C,E,F). However, MACC1 facilitated cell proliferation when glucose and glutamine are abundant (Figure 4B,C,E). Furthermore, MACC1 supported glutamine use reflected by enhanced cell viability of SW620 shcntl and HCT116 shcntl cells compared to their shMACC1 counterparts, if glucose is depleted and with a reduced glutamine concentration in a physiological range of 0.5 to 1 mM (Figure 4D,G). Out of these cell proliferation/viability studies, 10 mM glucose/2 mM glutamine and 0 mM glucose/1 mM glutamine conditions were considered to be positive hits.

Next, we analyzed if MACC1 influences glutamine consumption in SW620 shcntl and SW620 shMACC1 cells. Under 10 mM glucose/2 mM glutamine conditions MACC1 had no effect on glutamine depletion from cell growth medium, as demonstrated previously by GC-MS screening (Figure 4H). However, also under 0 mM glucose/1 mM glutamine, where we did see strong different cell viability (Figure 4D). Thus, the MACC1 effect on glutamine depletion was only moderate (Figure 4I).

MACC1 did not alter glutamine uptake under 10 mM glucose/2 mM glutamine (Figure 4H). However, this did not exclude the option that MACC1 may affect glutaminolysis under these conditions. Therefore, we investigated the MACC1 effect on cell viability upon inhibition of glutamine-using enzymes and applied the inhibitor 6-diazo-5-oxo-L-norleucin (DON) to SW620 shcntl and SW620 shMACC1 cells. DON treatment similarly reduced cell viability of SW620 shcntl and SW620 shMACC1 cells (Figure 4J). Although this shows the importance of glutamine for SW620 cells; however, there is no MACC1-dependent effect on cell viability.

This leads to the conclusion that if both glucose and glutamine are available, MACC1 increases proliferation only at higher glutamine concentrations. When glutamine is not present in the environment, MACC1 is unable to compensate its inability to provide an advantage in cell viability. If glucose is depleted, MACC1 facilitates glutaminolysis to elevate cell survival partially through increased glutamine uptake.

### 3.5. MACC1 Promotes Pyruvate and Restricts Lactate Use in Glucose-Depleted Environment

**Figure 5:**
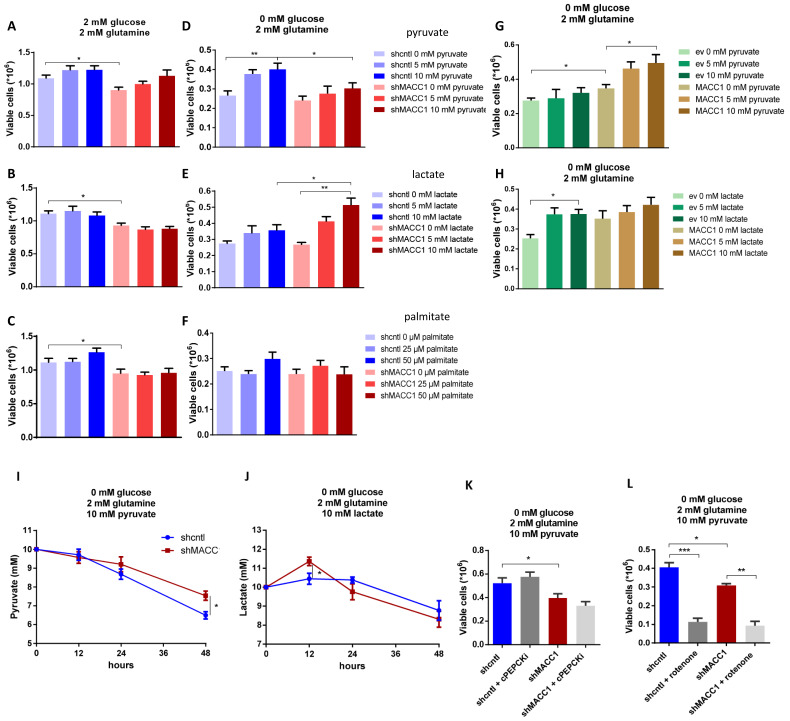
MACC1 promotes pyruvate and restricts lactate use in glucose-depleted environment. (**A**–**F**) Cell viability of pyruvate treated SW620 shcntl and shMACC1 cells, lactate and palmitate at indicated concentrations in (**A**–**C**) low glucose and (**D**–**F**) glucose-depleted conditions. (**G**–**H**) Cell viability of SW480 ev and MACC1 overexpressing cells treated as indicated in glucose-depleted conditions. (**I**) Pyruvate and (**J**) lactate depletion from cell growth medium of SW620 shcntl and shMACC1 cells after 48 h cultivation under indicated conditions. Cell viability of SW620 shcntl and SW620 shMACC1 cells treated at indicated conditions with (**K**) cytoplasmic phosphoenolpyruvate carboxykinase (PEPCK) inhibitor (cPEPCKi) and (**L**) mitochondrial complex I inhibitor rotenone. * *p* ≤ 0.05, ** *p* ≤ 0.01, *** *p* ≤ 0.001. Data represent mean values ± SEM of at least three independent experiments.

Pyruvate, lactate and fatty acids are known to aid cancer cell survival in nutrient stress conditions [17,19,20]. Thus, we investigated if MACC1 may provide a growth advantage when pyruvate, lactate and palmitate are used as alternative metabolites in low glucose (2 mM) and glucose-depleted (0 mM) environments. MACC1 did not increase proliferation due to use of any of the assessed alternative metabolic fuels in low glucose conditions (Figure 5A–C). In glucose-depleted conditions MACC1 enhanced growth with pyruvate (Figure 5D), restricted growth with lactate (Figure 5E), but had no effect on growth using palmitate (Figure 5F). This was confirmed in SW480 cells, where MACC1 overexpression increased proliferation when pyruvate was supplied. There were only minor effects on proliferation when lactate was supplied (Figure 5G–H). Conditions of 0 mM glucose/2 mM glutamine and 10 mM pyruvate were considered to be positive hits and taken for further work.

Next, pyruvate and a lactate depletion assay were performed to analyze an MACC1 effect. In SW620 shcntl compared to SW620 shMACC1 cells, pyruvate depletion was moderately increased (Figure 5I), while lactate use was not altered (Figure 5J).

Pyruvate can serve as substrate for gluconeogenesis and run glycolysis, or can be metabolized in the tricarboxylic acid cycle (TCA), to fuel the mitochondrial respiratory chain. To clarify which pathway is used by SW620 shcntl cells compared to SW620 shMACC1 cells, we inhibited either the gluconeogenesis or mitochondrial respiratory chain. Treatment of SW620 shcntl and SW620 shMACC1 cells with an inhibitor of cytoplasmic phosphoenolpyruvate carboxylase (cPEPCK) as first enzyme in gluconeogenesis, did not alter cell viability (Figure 5K). Thus, pyruvate did not enter the gluconeogenic pathway, or alternatively, the mitochondrial PEPCK isoform was prevalent. By contrast, treatment with the mitochondrial complex I inhibitor rotenone reduced cell viability; however, to the same extent in SW620 shcntl and SW620 shMACC1 cells (Figure 5L). This demonstrates the intrinsic property of mitochondria in SW620 cells, which is MACC1-independent in the context of pyruvate use.

Taken together, in a glucose-depleted environment MACC1 supports use of pyruvate and restricts lactate use through altered nutrient depletion. This defines cell nutrient preferences and provides an advantage for cell survival.

### 3.6. MACC1 Affects Central Carbon Metabolism in Multiple Ways

As MACC1 showed an impact on cell proliferation dependent on nutrient availability, it was of interest to investigate possible differences in nutrient allocation to cellular pathways. To address this question, growth medium which contained metabolic tracers uniformly labeled with ^13^C was used to cultivate SW620 shcntl and SW620 shMACC1 cells for 24 h followed by GC-MS-based metabolic profiling of cells and cell growth medium. To this end, we tested glucose, glutamine as well as pyruvic acid as suitable tracers in 3 independent experiments. Due to the long labeling time (24 h) steady state labeling can be assumed. Although medium composition was similar for glucose and glutamine experiments (containing 2 mM glutamine and 10 mM glucose), pyruvic acid tracing had to be performed under no glucose conditions to enhance tracer uptake. Only metabolites which are generated by cells from the respective tracer molecules will be enriched for ^13^C containing isotopologues and only those metabolites which were found to be enriched in any of the 3 parallel experiments are reported here.

**Figure 6:**
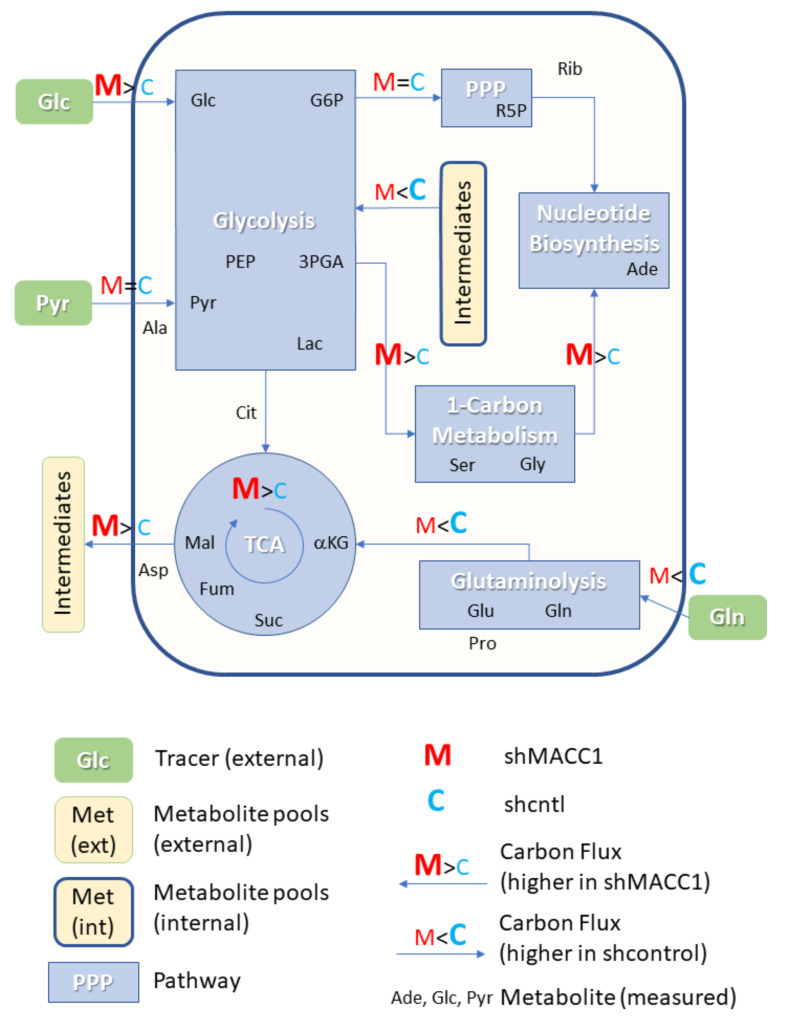
MACC1 dependent effects on cellular pathways. Overview of cellular pathways where MACC1 dependent effects could be monitored due to detectable differences in ^13^C tracer incorporation. Evidence for the depicted differences is provided in Figure 7 and the manuscript text. (M: shMACC1, C: shcntl, PPP: Pentose Phosphate Pathway, TCA: Tricarboxylic Acid Cycle, Glc: Glucose, G6P: Glucose-6-phosphate, 3PGA: Glyceric acid-3-phosphate, PEP: Phosphoenolpyruvic acid, Pyr: Pyruvic acid, Lac: Lactic acid, R5P: Ribose-5-phosphate, Rib: Ribose, Ade: Adenosine, alpha-, A: Adenine, Cit: Citric acid, aKG: Glutaric acid, 2-oxo-, 2HG: Glutaric acid, 2-hydroxy-, Suc: Succinic acid, Fum: Fumaric acid, Mal: Malic acid, Ala: Alanine, Pro: Proline, Asp: Aspartic acid, Asn: Asparagine, Gln: Glutamine, Glu: Glutamic acid, Gly: Glycine, Ser: Serine.).

In total 24 metabolites were monitored, which can be assigned to various pathways of the central carbon metabolism and allow conclusions on metabolic rewiring following MACC1 knockdown by comparing observable enrichment patterns from independent tracer experiments (Figure 6).

**Figure 7:**
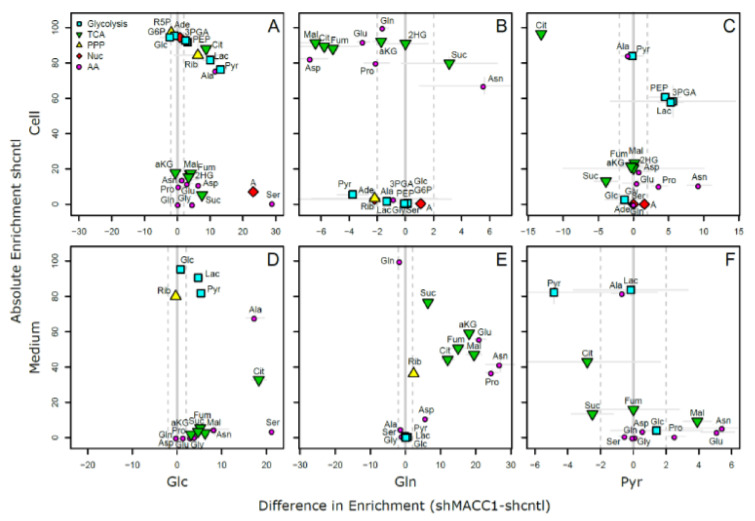
Metabolite labeling differences between shMACC1 and shcntl cells. Absolute labeling compared to labeling difference between shcntl and shMACC1 for intracellular (**A**–**C,** top panel) and medium (**D**–**F**, bottom panel) levels using Glc (**A**,**D**), Gln (**B**,**E**) and Pyr (**C**,**F**) as ^13^C tracer. Metabolites further away from the center (grey line) of a subplot are differentially labeled. Dashed grey lines indicate a 2% window of uncertainty, i.e., the technical reproducibility of determining an enrichment. Individual uncertainties generally depend on the compound abundance and stability and standard deviations are indicated per metabolite as thin grey lines. (PPP: Pentose Phosphate Pathway, TCA: Tricarboxylic Acid Cycle, Nuc: Nucleic Acid Biosynthesis, AA: Amino acids, Glc: Glucose, G6P: Glucose-6-phosphate, 3PGA: Glyceric acid-3-phosphate, PEP: Phosphoenolpyruvic acid, Pyr: Pyruvic acid, Lac: Lactic acid, R5P: Ribose-5-phosphate, Rib: Ribose, Ade: Adenosine, alpha-, A: Adenine, Cit: Citric acid, aKG: Glutaric acid, 2-oxo-, 2HG: Glutaric acid, 2-hydroxy-, Suc: Succinic acid, Fum: Fumaric acid, Mal: Malic acid, Ala: Alanine, Pro: Proline, Asp: Aspartic acid, Asn: Asparagine, Gln: Glutamine, Glu: Glutamic acid, Gly: Glycine, Ser: Serine).

To provide a comprehensive overview of labeling patterns within cells and in the medium determined in 3 assays using different tracers while at the same time highlighting the contrasts between shcntl and shMACC1 replicates, we devised a specific figure layout (Figure 7). Here, metabolites are color coded according to pathway association and depicted with respect to their absolute labeling (amount of ^13^C over total C within molecules) on the *y*-axis, while indicating the difference in absolute labeling between shcntl and shMACC1 on the *x*-axis. Metabolites with high values on the *y*-axis are therefore predominantly produced from the respective tracer (e.g., glycolysis intermediates in cell and medium samples with glucose as tracer). Metabolites deviating from zero on the *x*-axis are differentially labeled between shcntl and shMACC1. Negative values indicate higher labeling in shcntl and positive values indicate higher labeling in shMACC1. This layout allows the inference of several qualitative conclusions regarding the metabolic flux within cells.

The upper panel of Figure 7 shows that shMACC1 cells incorporate more glucose than shcntl cells but less glutamine with pyruvate being incorporated at comparable amounts. This is supported by the distribution of metabolites along the *x*-axis, showing the difference in absolute labeling between cell types. Here, we note that most metabolites show positive values for glucose labeling (Figure 7A), negative values for glutamine labeling (Figure 7B) and equally distributed values for pyruvic acid labeling (Figure 7C), supporting the previous statement. We further note that carbon supply into the TCA cycle is predominantly originating from glutaminolysis in both lines. This is apparent when comparing enrichment levels of TCA cycle intermediates (green triangles) in glucose and glutamine tracer supplied cells, where about 90% of the molecules are labeled using glutamine but only about 20% if glucose is provided as a tracer. Total labeling generally is a good indicator of the relative amount of a compound produced from the tracer. This is nicely seen for glycolysis intermediates in Figure 7A (blue squares) showing a slight decrease in total labeling along their expected order within the pathway. Although G6P (95% labeling) is nearly exclusively produced from glucose, pyruvic acid pools (80% labeling) are also resupplied from non-labeled sources by 20%.

Compared to shcntl cells, shMACC1 cells show a stronger secretion of TCA cycle intermediates, which is indicated by comparing their intra and extra cellular labeling states (Figure 7B,E). Although shMACC1 cells show about 5% less ^13^C incorporation, the secreted TCA cycle intermediates in medium samples of shMACC1 cells are about 20% higher labeled.

Other metabolic pathways, such as pentose phosphate pathway and nucleotide biosynthesis, which could be investigated due to observations in Rib5P and Adenosine respectively, do not show significant differences in overall labeling when MACC1 expression is knocked down. However, the detailed isotopologue distribution of Adenosine (Supporting Figure S1) shows systematic deviations with isotopologues M6 and M7 (i.e., where 6 and 7 carbon molecules are ^13^C or 1 and 2 additional to the 5 C from ribose respectively) being nearly exclusively present in shMACC1. This observation is likely connected to the most striking difference we observed: Serine is labeled >25% in shMACC1 cells but shows nearly no incorporation of ^13^C in shcntl cells (Figure 7A). Possible explanations could be that serine in shcntl cells is produced from different metabolic sources than glucose or taken up from the media. However, the latter hypothesis is less likely given that labeled serine is found in media of shMACC1 cells hinting at secretion of this metabolite. Together, these findings indicate strong remodeling of 1-carbon metabolism by MACC1.

Using ^13^C_3_ labeled pyruvate as a tracer we intended to monitor the potential for gluconeogenesis. To this end, metabolic profiling revealed that pyruvate derived ^13^C carbon was deposited in metabolites of gluconeogenesis, TCA cycle, amino acids and nucleotides, whose synthesis is closely linked to TCA cycle metabolites (Figure 7C). As with ^13^C_6_ glucose and ^13^C_5_ glutamine labeling experiments, numerous pyruvate derived metabolites, which were found intracellularly, were also present in the medium (Figure 7F). Interestingly, ^13^C_3_ labeled pyruvate was found to be converted to lactate, phosphoenolpyruvate and glycerate, where the latter two metabolites are substrates for the gluconeogenesis. The generated ^13^C labeled glucose was found only in trace amounts. Although glutamine feeds the TCA cycle, generation of glycolytic/gluconeogenic intermediates from pyruvate guides additional carbon to those metabolites, which are critical for generation of biomass. This compensates lack of glucose and thus explains the increased cell viability in the presence of pyruvate.

Citrate molecules within cells are predominantly derived from pyruvate (Figure 7C) and citrate derived carbon is known to fuel the TCA cycle. However, in both glucose and pyruvate labeling experiments, TCA cycle intermediates were only labeled to about 20% indicating strong glutaminolysis.

Overall, we could show that MACC1 knockdown leads to differential metabolite labeling in various parts of the central metabolism, probably due to the differential use of glycolysis and glutaminolysis, which can be expected to contribute to the observed growth differences.

## 4. Discussion

The ability of an oncogene to affect nutrient use not only depends on nutrient availability in the microenvironment, but also on cellular ability to take up and use the nutrient within metabolic pathways, thus gaining a metabolic source to satisfy ever growing demands for energy and cellular building blocks. For characterization of cancer metabolism several approaches and techniques are available [43]. Most of them were applied in this work to better understand the role of MACC1 in CRC metabolism. This study aimed at analysis of the impact of MACC1 on the use of glucose, glutamine, pyruvate, lactate and fatty acids. Our results demonstrate that MACC1 context-dependently supports use of glucose, glutamine and pyruvate.

First, we evaluated the impact of MACC1 on glucose consumption. Interestingly, increased surface GLUT1 expression potentially enhances glucose uptake and therefore may increase cell proliferation. The ability of MACC1 to increase surface GLUT1 shows the potential of MACC1 to mediate translocation of GLUT1 from the endosomal pool to the cell surface. PI3K/Akt signaling is a known main trigger of GLUT1 shift to the cell surface [44,45]. MACC1 is linked to increased PI3K/Akt signaling and pharmacological Akt inhibition leads to reduced MACC1 expression in gastric cancer [31]. This indicates a feedback loop between MACC1 and PI3K/Akt signaling. Furthermore, we suggest a positive feedback loop between MACC1, GLUT1 and glucose. The increase in glucose depletion by MACC1 [30,31] suggests a MACC1-mediated effect on glucose depletion which is present in CRC and gastric cancer. 

The serine synthesis from glucose by MACC1 knockdown was an important finding from the glucose flux experiment. Serine is an important metabolite, which contributes to one-carbon metabolism, important for folate and amino acid metabolism, biosynthetic processes, epigenetic modifications and redox balance [46]. Metabolic flux studies showed that cancer cells may use up to 50% of glucose-derived carbon for serine synthesis [47], and that serine is a big contributor to NADPH production [48]. The generation of manifold one-carbon tetrahydrofolate species from serine protects cells from hypoxia-induced oxidative stress [49]. Thus, MACC1 might provide metabolic advantage to cells for adaptation to various metabolic and nutrient environments to ensure cell survival.

Metabolic functional studies revealed that MACC1 reduced mitochondrial respiration, whereas MACC1 effect on extracellular acidification was inconsistent. One of the reasons of this observation may be that the acidification should not be treated as pure readout for glycolysis, since besides lactate derived from glucose, bicarbonate produced in high amounts by actively respiring mitochondria can also contribute to it. Thus, contribution to acidification depends on the ratio between glycolytic activity and mitochondrial respiration. If the amount of bicarbonate produced by the mitochondria is higher than the amount of lactate contributing to acidification, cells will have higher acidification without being glycolytic.

Since pyruvate is an intermediate of glycolysis, it is not surprising that MACC1 supported pyruvate use only upon glucose depletion. In breast cancer, inhibition of MCT1 in MCT1 and MCT4 co-expression cells led to decreased pyruvate, but not lactate export. This demonstrates that besides lactate, glycolytic cells also export pyruvate, thereby feeding starving nearby cells [50]. This phenomenon is also known as metabolic symbiosis, which contributes to resistance toward targeted therapies [51,52]. The fact that MACC1 supports pyruvate but not lactate use demonstrates its ability to shape cell nutrient preferences. Unlike in breast cancer, where pyruvate is used via TCA cycle [18], CRC cells have a stronger demand in glycolytic/gluconeogenic intermediates and therefore use pyruvate via TCA cycle and also gluconeogenic pathway.

The metastatic process requires adaptation to varying environmental conditions, which migrating cells encounter on their way to a suitable soil for cell attachment, invasion, adaptation and growth. MACC1, a key driver of metastasis, demonstrates the ability to affect nutrient depletion both in vitro and in vivo, thus bringing more carbon to create building blocks necessary for cell growth and survival. PGC-1α, a well-established regulator of cancer metabolism, has been reported to promote metastasis. This ability was associated with increased bioenergetics capacity giving implications to resistance to metabolic drugs [53]. This demonstrates that the ability of metastatic cells to adapt and grow at distant locations is accompanied with altered nutrient depletion and use. Accordingly, MACC1 expressing CRC tumor lesions and metastases revealed an increased uptake of ^18^F-FDG and ^18^F-glutamate in our in vivo mouse experiments. On the other hand, glucose-mediated induction of MACC1 expression and the fact that MACC1 expression is increased at the tumor invasive front [54], where glucose availability is higher than in the necrotic tumor center, may explain the increased invasiveness of MACC1 expressing cells.

The ability of an oncogene to rewire cell metabolism and to alter nutrient uptake is common among many tumor entities [6]. Different oncogenes had been demonstrated to induce different metabolic profiles. In prostate cancer Akt1 overexpression is associated with accumulation of glycolytic metabolites, whereas overexpression of MYC causes dysregulation in lipid metabolism [3]. MYC increases catabolism of glucose and glutamine in liver cancer, while MYC-driven lung tumors show increased expression of glutamine synthetase and glutaminase. MET-driven liver tumors use glucose to produce glutamine [4]. This work revealed MACC1-driven metabolic networks in normal and stressed conditions, which may allow the design of improved therapies for treating MACC1 expressing tumors and their metastasis.

## 5. Conclusions

In summary, the findings of this study demonstrated that MACC1 is a novel and potent regulator of cancer metabolism exerting multiple effects on metabolic rewiring. The ability of MACC1 to enhance metastasis is accompanied with altered nutrient use by either altered nutrient depletion or metabolic flux in normal and stressed conditions. By this, MACC1 contributes to metabolic flexibility of the cells in adaptation to the environmental stresses and thus ensuring cell survival and metastatic outgrowth.

## Data Availability

The data presented in this study are available on request from the corresponding author.

## Supplementary Materials

The following are available online at https://www.mdpi.com/2072-6694/13/5/978/s1, Figure S1: Mass isotopomer distribution, corrected for the occurrence of natural abundant 13C.
