# Supplementary figures and images for: Systematic Identification of MACC1-Driven Metabolic Networks in Colorectal Cancer
Source: Cancers (Basel). 2021 Feb 26;13(5):978. doi: 10.3390/cancers13050978 (PMC7956336; doi:10.3390/cancers13050978)

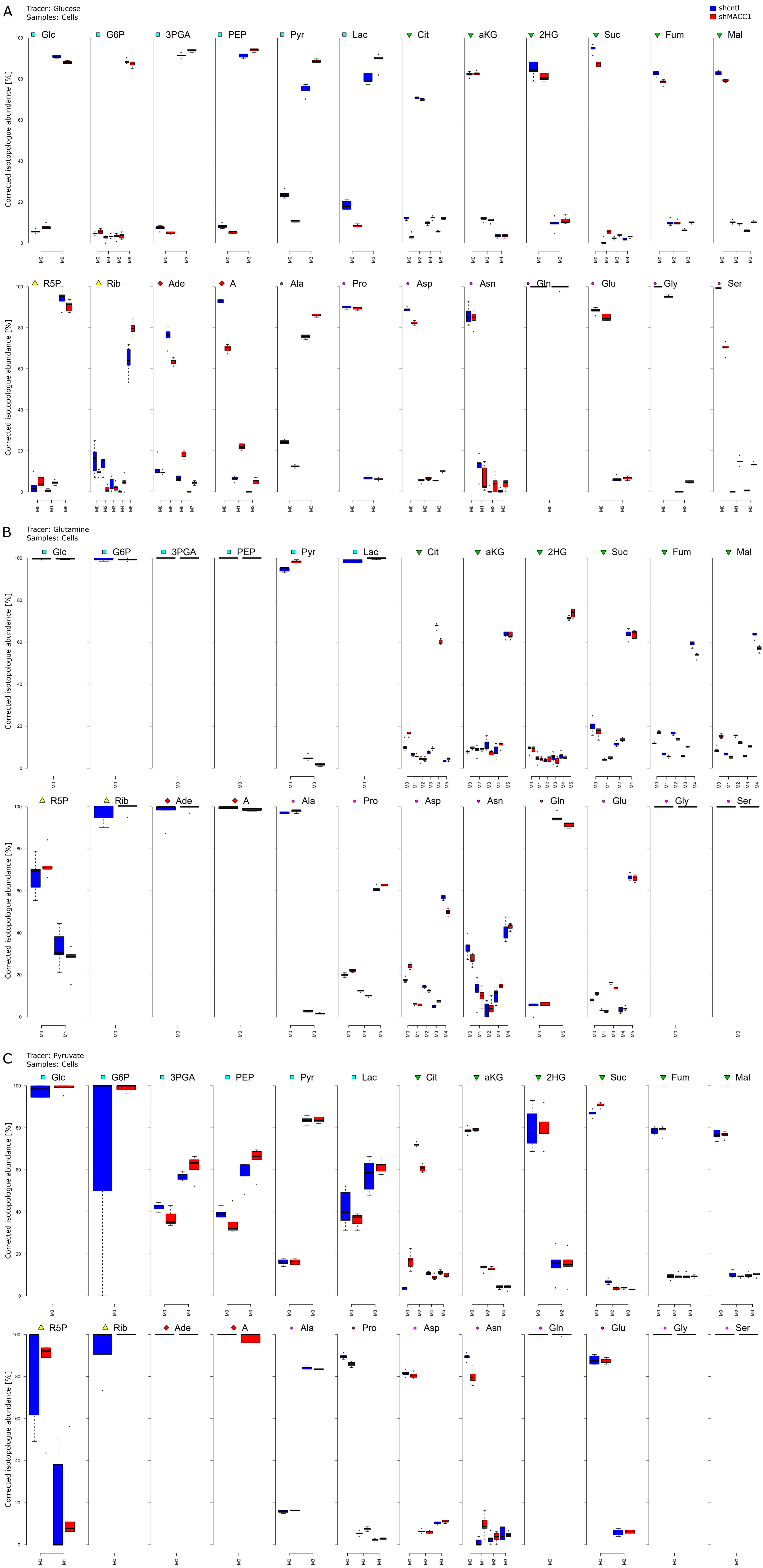

Supplement: Supplementary file 1 [file cancers-13-00978-s001.pdf]
